# The public's acceptance of novel vaccines during a pandemic: a focus group study and its application to influenza H1N1

**DOI:** 10.3134/ehtj.09.008

**Published:** 2010-03-26

**Authors:** N Henrich, B Holmes

**Affiliations:** 1 Centre for Health Evaluation and Outcome Sciences - St Paul´s Hospital, Vancouver, British Columbia, Canada; 2 Simon Fraser University -School of Communication, Vancouver, British Columbia, Canada

## Abstract

As influenza H1N1 spreads around the world, health officials are considering the development and use of a new vaccine to protect the public and help control the outbreak. Acceptance of novel vaccines during health crises, however, is influenced by perceptions of a range of risks, including the risk of infection, risk of becoming severely ill or dying if infected, as well as the risk of serious side and long-term effects of the vaccine. A study on 11 focus groups was conducted with the public in Vancouver, Canada in 2006 and 2007 to explore how people assess these risks and how these assessments relate to their willingness to use novel vaccines in a pandemic. Concerns about using new vaccines during a pandemic differ from concerns about using established products in a non-crisis situation. Participants were hesitant to use novel vaccines because of a low perception of the early risk of infection in a pandemic, coupled with the many uncertainties that surround new vaccines and the emerging infectious disease, and owing to the concern that unsafe pharmaceuticals may be rushed to market during a health crisis. Understanding the public´s assessment of the risks related to, and willingness to use, novel vaccines during a pandemic can help officials promote disease-control measures in ways that improve the likelihood of acceptance by the public and may increase uptake of an H1N1 vaccine.

## Introduction

Public health officials have been worried for some time about the imminent threat of an influenza pandemic, either the influenza A H5N1 virus or, most recently, the H1N1 virus.^1^, ^2^ Although a specific pandemic virus could not be anticipated with certainty, it was important to understand how the public may react to a pandemic in advance of the crisis and then apply the findings during an actual outbreak, at which point public health emphasis focuses on disease control and treatment rather than on qualitative research with the public. One question addressed during the pre-pandemic period was how the public may perceive new vaccines for use in a pandemic. At present, as health agencies, the WHO and drug companies work together to develop a vaccine for the H1N1 virus,^3^, (4) we can draw on this research to answer the question: What influences a person's decision to get vaccinated with a novel vaccine in the event of a pandemic? Assuming a vaccine is developed, a campaign to vaccinate all or large segments of a society will be effective only if the public is willing to get vaccinated.

Decision making regarding the use of vaccines is, in part, influenced by how the public assesses the risks associated with a disease (how likely they are to become infected and how sick they may become if infected) and the vaccine. Little is known, however, with regard to the manner in which the decision-making process is affected by the uncertainties associated with a new disease and new vaccines that would be developed to control it. These uncertainties are especially great early on in a pandemic when very little may be known about the disease, about at-risk populations and about the effectiveness and safety of control mechanisms. In this case, understanding how vaccination decisions may be made in the event of a pandemic will help to develop strategies to increase the likelihood of compliance with vaccination recommendations.

## Methods

A study on 11 focus groups with a total of 85 members of the public was conducted in Vancouver, BC, Canada between November 2006 and June 2007. The goal of the study was to ascertain the attitudes, concerns and perceptions regarding the risk of emerging infectious diseases (EIDs) and the use of new vaccines. Focus groups were the chosen methodology because the format allows for broad discussions and interactions that can hone the development of topics for qualitative surveys with larger populations. Ethics approval for the research was received from the University of British Columbia's ethics review board.

### Participants

Participants for the focus groups included the following, selected to represent a broad spectrum of opinions and perspectives:

- two groups of university students;
- three groups of adult Canadians, including one group each from the Chinese community, new immigrants and non-ethnic Canadians;
- three groups of parents: two groups known to be skeptical of, or opposed to, childhood vaccinations because of their non-mainstream or ‘alternative’ beliefs—we refer to these as ‘alternative’ parent groups; one group from a ‘mainstream’ community that is believed to have societally predominant attitudes about childhood vaccinations—we refer to this group as ‘mainstream’ parents;
- three groups of health-care workers (HCWs): this group included any health-care professional working for the Vancouver Coastal Health Authority, with participants representing a diverse array of occupations such as nurses, physiotherapists, social workers, health administrators and health educators.

Note that participants in any of the groups could be parents, but all participants in the parent groups were required to be parents in order to participate in those sessions.

The Chinese community was targeted because Chinese people make up 43.6% of the visible minority population in Vancouver and 19.2% of Vancouver's total population. No other visible minorities constitute nearly as large a percentage of the population.^5^ Participants in this group self-identified themselves as Chinese and were recruited from a Chinese community center. The new immigrant group was included in the study because we speculated that recent immigrants from countries outside of Western Europe and North America may not be acculturated into, or fully accessing, the biomedical health-care system and consequently may be less likely to use vaccines.^6^–^9^

University students were included because of the importance of vaccinating students during an outbreak, given this population's high number of close social contacts both in the classroom and in their group living environments. As students’ decisions often diverge from those of non-student adults in their populations, 10–14 we could not assume that information obtained from non-student adults would reflect students’ perspectives.

Parents were targeted because they would be making decisions regarding vaccination for their children, as well as for themselves. The ‘alternative’ parents were included because it is known that alternative healthcare providers (for example, homeopaths, naturopaths and chiropractors) tend to recommend that their patients refuse vaccinations,^15^–^17^ and this population could pose a threat to the public's health if they refuse to vaccinate themselves or their children during a pandemic. The ‘mainstream’ parents served as a control for the ‘alternative’ parents and represented the majority of parents in the population.

HCWs were included because they would be involved in vaccinating people during a pandemic and, potentially, in educating patients about the vaccines.

### Recruitment

Participants were recruited with an email and/or flyer. One group of students was also recruited with an announcement made in a large, multi-discipline undergraduate class and one group of ‘alternative’ parents was recruited by posting our recruitment email on a parent listserv known to be used by parents who have non-traditional attitudes about health and other social issues. HCWs were recruited by sending a recruitment email to all employees of the Vancouver Coastal Health Authority.

All participants, except HCWs, received an incentive of $25, which was equivalent to approximately double the local minimum wage at the time of the study. HCWs received $40, which was calculated by averaging a typical hourly wage for nurses and new physicians.

### Procedure

Each focus group discussion lasted approximately one and a half hours and was audio taped. At the beginning of each session, the leader described a fictitious disease. Participants were informed that a new, sometimes deadly, disease had developed in India that caused symptoms including bloody diarrhea, severe headaches, muscle and joint pain, and as the disease progresses, difficulty in breathing, and that the disease was spreading from person to person but had not yet spread outside of India. It was stated explicitly that this was a fictitious disease. The group was then asked questions regarding (1) their perceptions of risk as the disease spread around the world and eventually arrived in Vancouver, and (2) their concerns, questions and attitudes about, and willingness to use, hypothetical new vaccines developed to prevent the disease. The issue of how the novelty of the disease and the vaccine may impact attitudes and behaviors was queried in each group.

### Analysis

Focus group recordings were transcribed and uploaded to *QSR-Nvivo7* for coding and qualitative analysis. A two-pronged approach was taken for the coding. One set of codes was selected on the basis of research questions, capturing topics that we determined *a* *priori* and that we wanted to learn about. A second set of codes captured themes and concepts emerging in the discussions that we did not necessarily anticipate. The codes were developed and reviewed by both researchers. All coding was carried out by one individual to ensure consistency, and the coded transcripts were reviewed by the other researcher. Analyses were performed by reviewing all conversations within a given code or cluster of codes for a topic. Each focus group was reviewed separately so that differences across focus group populations could be detected. Frequencies of discussion content were tracked to determine which of the comments, attitudes, concerns, etc. were expressed most frequently and which were rare, and to assess whether a different content was expressed with differential frequency across groups. Conclusions were drawn on the basis of the content and frequency of the discussion comments for a topic.

## Results

The findings from the focus groups presented in this section represent opinions that were commonly expressed across focus groups. If a finding/quotation characterizes a minority opinion or occurred in only one or a few of the groups, it is specifically indicated.

### Risk of infection

#### Proximity and ease of spread

When the disease was still confined to its country of origin, most participants said they would have minimal concern about their personal risk of infection and many would simply monitor the disease's progress. Representative comments include:

I would be interested but I wouldn't be worried. Like I'd follow it but I wouldn't be worried, worried. I'd be interested to find out what happened, I guess. [Student] I would stay informed actually…. My awareness would be heightened. [‘Alternative’ parent]

In all groups, except for the ‘alternative’ parents, at least some individuals felt they would be at risk if the disease was only in its country of origin. In the new immigrant group, one of the student groups and one of the HCW groups, all but one participant in each group felt they would be at risk. In contrast, of the participants in the two groups of ‘alternative’ parents, only one person felt at-risk (others felt either no risk or monitored the situation)

People who felt at-risk tended to focus on the great amount of international travel that goes on today and several people made reference to other diseases that have spread rapidly around the world.

Because the, I think if the illness can spread easily from one country to another country. Like AIDS. I remember that AIDS, first time came from monkey also. And, then spread it very fast in all continents and countries and also SARS and Avian Flu. [New immigrant]

Among those who did not have any concern about the disease when it was contained in its country of origin, explanations included references to other diseases that emerged outside Canada and never reached here, and the possibility that, as the disease had not yet left India, perhaps it never would.

Well the Avian Flu has been in the Asian countries for a number of years now, and I don't think there has been a recorded case here in [Canada]. [HCW]

Overall, people's level of perceived risk of infection increased as we described scenarios in which the disease moved ever closer to home. This was captured succinctly by an HCW, who said, ‘The more it spreads, the more my concern would increase. It would just be a direct ratio.’ Although many were adamant that they would not panic or feel a high level of personal risk, a few people said that they would begin to feel alarmed when the disease arrived in BC. For parents, panic or great apprehension tended to relate to concerns about their children's health.

#### Immunity

Unlike participants in other groups, the ‘alternative’ parents remained largely unconcerned about the disease, even as it spread. Only two of these parents were worried about being infected, even as the disease arrived in North America. The primary reason for these parents’ lack of concern was their belief that being in good health and eating well would give them a strong immune system that prevents infection.

I also think that—I heard this quote that Louis Pasteur on his deathbed said, ‘the microbe is nothing, the terrain is everything’ and I would be really like getting rid of the sugar okay…a super healthy immune system would be the way I would want to go. [‘Alternative’ parent]

Although it was mentioned less often outside of the ‘alternative’ parents group, other participants raised the issue of the strength of a person's immune system. These people were not worried about contracting the disease because they were healthy and because they believed that the disease would probably affect only elderly people and others with compromised immune systems.

#### Personal control

In all the groups, there were participants who believed that they could adopt measures to protect themselves from getting infected, such as handwashing and staying away from crowded places and sick people, and for HCWs, by following ‘universal precautions’ such as wearing masks and being cautious of needles. The strong belief that people can mitigate, if not control, the risk of becoming infected was expressed in comments such as the following:

I have faith. I have faith in our personal hygiene … [Non-ethnic Canadian]

A lot, well all of us probably practice basic body, blood, fluid precautions, right?...So, we're equipped in that way to handle new diseases. We know how to protect ourselves. At least we think we do. [HCW]

#### Information and media

Participants´ perception of the risk of infection is largely influenced by the information they receive about the disease. Many of the participants stated that having information about factors such as modes of transmission, groups of people who are at greatest risk of infection, severity of the illness and risk of death, and how the disease can be prevented or treated would enable them to make better informed decisions and give them a sense of control. In contrast, uncertainty breeds fear and an elevated perception of risk. As a non-ethnic Canadian explained, ‘I think the less information the more hysteria you have around the whole issue. So, the more information, the better you're able to analyze the risks.´

It was suggested by participants in seven focus groups that the media could increase fear about the new disease simply because they would be covering the story extensively. Constant coverage of the situation would lead people to assume that the threat was highly independent of any other facts about the disease or the pandemic. As a ‘mainstream’ parent said, ‘If it's something that was constantly in the media, I probably would be more concerned about it than other things just because of that perception, that's it a more dramatic thing, I think.’ Some participants believed that the media would hype the situation and induce greater fear. These sentiments were expressed in the following sessions: two ‘alternative’ parent groups, the ‘mainstream’ parent group, two student groups, one HCW group and the nonethnic Canadian group. The new immigrant, Chinese community and two of the HCW groups did not mention it, although all but the new immigrant group raised issues about unreliability or mistrust of the media.

#### Novelty of disease

Participants generally shared the belief that they would feel more threatened by an emerging disease than by an established one. This elevated risk perception was due to the many unknowns of a new disease, the susceptibility of the population to infection, as well as because of a lack of prevention and treatment measures. People seem to associate a danger with an EID that they do not feel for diseases that have afflicted our population for many years. References were made, for example, to the greater threat of an EID than of tuberculosis or chicken pox.

### Use of novel vaccines

In the context of the fictitious disease, participants were asked whether, if a new vaccine were developed, they would get vaccinated or have their children vaccinated. Very few people said they would definitely get vaccinated.

#### Safety

The most influential factor in determining vaccination is the safety of a new vaccine, especially one that is developed rapidly in response to a health crisis. Participants were greatly concerned that, in a pandemic, a vaccine would be brought to market without sufficiently testing for safety.

But the one thing that I have never understood about, you know, this idea of developing vaccines for a new or emergent threat, how do they have time to go through all the testing that they usually go through for vaccines if it is such—um, like, an immediate sort of need. How do they do that and do they do that? [´Alternative´ parent]

Participants were extremely hesitant to be the first users of a product. There was a shared belief that there could be problems with the safety of the vaccine that would only surface after it has been used by many people and with sufficient time for long-term effects to emerge. Across the focus groups, participants felt that if they used a novel vaccine they would be guinea pigs.

And I think that just given that the short time frame, whatever a year, in which this disease has emerged, I would be really quite wary of how they'd be able to have significant clinical trials in that amount of time and have proven that there aren't any side-effects in 10 years, 5 years. You never know till your children. So I would be kind of wary about that. [Student]

Hesitancy with regard to getting vaccinated with a new vaccine does not reflect an overall distrust or dislike of vaccines; wariness was related specifically to the novelty of the vaccine.

#### Severity of morbidity from infection

In conjunction with considering vaccine safety, focus group participants said they would base vaccination decisions largely on the severity of morbidity if they were to become infected. When deciding whether to get vaccinated, people basically want to know how sick or impaired they would become from the vaccine and whether they would become severely ill or die if they get the disease. Participants said they would weigh information about issues such as how long they would be sick and how debilitating the illness is against the possibility and severity of side and long-term effects from the vaccine. These sentiments were captured by an ‘alternative’ parent who said that in order to make a vaccine-use decision she would want to know about:

Side-effects, any sort of long-term side-effects that they might think—I guess the pros and cons versus using it and not using it. I think probably would want to know a lot more about the disease as well, not specifically just the vaccine, but find out as much as possible about the disease, the severity and longevity.

Similarly, an HCW explained: ‘Information would be key and I'd have to weigh the cost and the benefit of…. I'd have to know what would be the implications of getting the disease. And what would be the implications of getting the vaccine.’

#### Transmission

Part of the consideration for vaccination depends on the mode of disease transmission, with people being most willing to use a vaccine for an airborne disease. For respiratory transmission, people felt that they have no way to protect themselves, hence the risk of infection is greater than that with other modes of transmission. In contrast, people felt they have a lot of control regarding whether they could contract a disease that spreads sexually, and even HCWs were not overly concerned about blood-borne diseases because they could protect themselves using proper precautionary measures.

#### Children versus adults

Parents in the focus groups assessed the risks of novel vaccines and EIDs differently for themselves than for their children. A minority of parents said that they would prioritize vaccinating their children, especially if the disease could cause serious morbidity or death. Overwhelmingly, parents claimed that they were more likely to vaccinate themselves than their children. This reflects their belief that the vaccine's side-effects, and especially the long-term effects, pose a greater risk than the disease. They were willing to take on the risk of vaccinating themselves but felt that it was more prudent to withhold the vaccine from their children. Particularly with long-term effects, parents indicated that because the children were young, there were many more years for currently unknown long-term effects to develop in their children than in themselves.

I guess I have more responsibility for [my child] and his well being and his life. I don't know. I guess I am just a little bit more blasé with things I do to myself than I might do to him. Things that I take for myself that I might not do for him. [‘Alternative´ parent]

I think it would be different for me to vaccinate myself rather than my children. Because I mean, I feel like, I feel like, you know, I'd probably have a better immune system. But to expose my children to something that is absolutely brand new, I don't know. I would hesitate. [‘Mainstream´ parent]

The ´mainstream´ parents were not generally opposed to vaccinating their children; eight out of the nine parents in this group had had their children vaccinated. However, they perceived the risks of a novel vaccine as being much greater than those for the standard set of childhood vaccines that have been around for years. The ´alternative´ parents tended to be wary of any vaccines—old or new—and their unwillingness to vaccinate their children with the new vaccine reflects their overall rejection of vaccines. Among the ´alternative´ parents, three had had their children vaccinated with all the recommended vaccines and the rest had either had some of their children partially vaccinated or none at all.

#### Alternative medicine

Some participants´ decision to use novel vaccines was influenced by the recommendations of alternative health professionals. These people sought alternatives to vaccines, which reflects their general preference for ´natural´ treatments rather than a specific rejection of novel vaccines. The influence of alternative health professionals was particularly prevalent among the ´alternative´ parent groups, who consult homeopaths or naturopaths for alternatives to biomedical products and seek their advice on which products to use. They also consult alternative health professionals and nutritionists to help maintain good health so as to avoid infection by EIDs.

Participants in the Chinese community and new immigrant groups, as well as one person in the ´mainstream´ parent group, also said that they prefer to explore alternatives (in the form of Chinese medicine or naturopathy). Individuals who consult alternative health professionals would not reject biomedical treatment, but they would not necessarily use it first or they may use it on the recommendation of their alternative health professional.

#### Pharmaceutical companies

A theme that recurred across the focus groups was not specific to novel vaccines but rather to vaccines in general—the motivation of the vaccine developers and of the person conducting the research on the safety and efficacy of the vaccine. Specifically, people expressed a distrust of vaccines developed by pharmaceutical companies because these companies may be motivated more by money than by public health, which could lead to the marketing of vaccines that are not safe, are ineffective or are just not really needed. Similarly, some participants were more willing to use vaccines that had been tested by independent researchers rather than those that were funded by a pharmaceutical company. People did not say that they would reject vaccines developed and tested by pharmaceutical companies, but rather that they would have heightened concern. This concern regarding pharmaceutical companies was raised by a group of ´alternative´ parents, both student groups, the ´mainstream´ parent group, two of the HCW groups and by the non-ethnic Canadian group. One ´mainstream´ parent and one HCW defended the pharmaceutical companies and the quality of their products.

## Discussion

The 2009 H1N1 outbreak began near the tail end of the usual flu season. It is speculated that this strain of flu will return during the next flu season.^18^ This provides a brief window of opportunity to produce and administer a vaccine for the disease. Drawing on the findings from the focus group discussions about the use of novel vaccines during a pandemic, it can be anticipated that public health officials will face several challenges in promoting vaccine uptake for H1N1. First, given that the public believes that they have control over whether they could become infected, it will be necessary to promote the use of personal control measures, such as frequent handwashing, covering your mouth when coughing or social distancing, while at the same time making it clear that these important preventive measures are not sufficient and that vaccination is still necessary and beneficial both for individuals and for the community.^19^

Second, the public wants information about the disease and the vaccine in order to make informed decisions about vaccination. However, making this information widely available needs to be balanced against providing too much media coverage to the pandemic, which can incite fear simply because frequent coverage is perceived to mean that the situation is grave.^20^

Third, it is imperative to communicate with alternative health professionals and discuss the merits and risks of vaccination for protecting individuals and controlling the pandemic. The inclusion of alternative health professionals is especially important because they often discourage patients from using vaccines.^16^, ^21^, ^22^ This segment of the health profession should not be overlooked; in the United States, for example, approximately 57% of the population uses alternative therapies and 10% receives services from alternative health-care providers;^23^ thus, the influence on their patients can mean the difference between whether or not herd immunity is achieved.

Fourth, given the high degree of suspicion expressed by focus group participants about the integrity and trustworthiness of pharmaceutical companies, vaccines may be more likely to be accepted by the public if they are developed in conjunction with academic institutions or the government.

Finally, it is important to identify and address the diverse concerns and perspectives of different groups in a population. 20 Targeted communication strategies that address the specific needs and attitudes of different segments of the population may lead to greater vaccine acceptance than a one-size-fits-all message. For example, on the basis of the focus groups, we identified the ‘alternative’ parents’ low perception of their risk of infection and their strong belief that boosting their immunity would provide protection that could make vaccination unnecessary, whereas other parents had specific concerns about how the long-term side effects of vaccines could affect their children. Ideally, subgroup differences should be identified before initiating a vaccination campaign so that communications can be targeted right from the beginning, rather than as a means of boosting vaccination rates belatedly in groups that vaccinate at low rates.

### Limitations of the study

1. The focus group participants are not representative of all members of the population to which they belong, and consequently the results are not generalizable.
2. Although efforts were made to recruit men and women, participants were mainly female (n_females_=72 (85%); n_males_=13 (15%)). Given that it is the women who generally make health decisions for their families,^24^–^26^ especially with respect to children and their vaccinations, the information obtained from the discussions may still reflect how households respond to an EID pandemic.
3. Data were collected during a non-crisis period and anticipated risk assessments may differ from those made during a pandemic. Our findings most likely mirror actual responses to risks in the early stages of a pandemic when people may perceive their risks from the disease as low.

### Strengths of the study

1. The study had a novel focus by targeting adults to explore their attitudes about self-vaccination, rather than the typical focus on either adults’ attitudes about vaccinating their children or the elderly's attitudes about self-vaccinating (thus capturing attitudes from a vulnerable population). Although studies have been conducted on HCWs’ attitudes toward self-vaccination, they have not been carried out in the context of a pandemic (see the next point).
2. The study focused on *new* vaccines for use against a *new* disease. As perceived risks vary with uncertainty, it cannot be assumed that findings from studies on attitudes about established vaccines for existing diseases can be extrapolated to pandemic situations. Our findings suggest that many vaccine attitudes from non-pandemic contexts can be applied in a pandemic, and also highlight concerns that are specific to the novelties of a pandemic situation. The unanticipated findings include the extent to which parents said they would vaccinate themselves but not their children, the perception that personal control measures can be perceived to be sufficient protection in a pandemic, thus minimizing the perceived need for a vaccine, and the widely shared concern that vaccine safety is compromised during a pandemic.
